# Different Types of Aortic Valve Stenosis in Patients Undergoing Transcatheter Aortic Valve Replacement

**DOI:** 10.1016/j.shj.2026.100817

**Published:** 2026-02-10

**Authors:** Kimberley I. Hemelrijk, Hugo M. Aarts, Gijs M. Broeze, Antonio Gomez Menchero, Didier Tchétché, Fabio S. de Brito, Marco Barbanti, Ran Kornowski, Azeem Latib, Augusto D’Onofrio, Flavio Ribichini, Miguel Artaiz Urdaci, Nicolas Dumonteil, Alexandre Abizaid, Samantha Sartori, Stefano Rosato, Giuseppe Tarantini, Gabriele Pesarini, Katia Orvin, Matteo Pagnesi, Beatriz Vaquerizo, George Dangas, Roxana Mehran, Astrid C. van Nieuwkerk, Ronak Delewi

**Affiliations:** a Department of Cardiology, Amsterdam UMC, University of Amsterdam, Amsterdam Cardiovascular Sciences, Amsterdam, The Netherlands; b Department of Cardiology, University Medical Center Utrecht, Utrecht, The Netherlands; c Cardiology Department, Hospital Universitario Juan Romón Jiménez, Huelva, Spain; d Department of Cardiology, Clinique Pasteur, Toulouse, France; e Heart Institute, University of São Paulo Medical School, São Paulo, Brazil; f Facoltà di Medicina e Chirurgia, Università degli Studi di Enna “Kore”, Enna, Italy; g Cardiology Department, Rabin Medical Center, Petach Tikva, Israel; h Department of Cardiology, Montefiore Medical Center, New York, New York, USA; i Division of Cardiology, Department of Medicine, University of Cape Town, Cape Town, South Africa; j Division of Cardiac Surgery, University of Padova, Padua, Italy; k Division of Cardiology, Department of Medicine, University of Verona, Verona, Italy; l Department of Cardiology and Cardiac Surgery, Clinica Universitaria de Navarra, Pamplona, Spain; m The Zena and Michael A. Wiener Cardiovascular Institute, Icahn School of Medicine at Mount Sinai, New York, New York, USA; n Global Health Department, Istituto Superiore di Sanità, Rome, Italy; o Institute of Cardiology, ASST Spedali Civili, Department of Medical and Surgical Specialties, Radiological Sciences and Public Health, University of Brescia, Brescia, Italy; p Cardiology Department, Hospital del Mar, Barcelona, Spain

**Keywords:** Aortic stenosis, Low gradient, TAVR

## Abstract

**Background:**

Transcatheter aortic valve replacement (TAVR) is an effective treatment in patients with “classical” concordant high-gradient aortic stenosis (AS). However, data on outcomes in patients with discordant AS are scarce. Our study aims to investigate the clinical outcomes of patients undergoing TAVR with different types of AS.

**Methods:**

The Cerebrovascular Events in Patients Undergoing Transcatheter Aortic Valve Implantation 2 (CENTER2) study is a patient-level database including 25,771 patients who underwent TAVR, of whom 15,233 were included in this analysis. Four AS subgroups were identified, and patients were classified as discordant AS (low-gradient AS with preserved or impaired left ventricular ejection fraction [LVEF] or discordant high-gradient AS) or concordant high-gradient AS.

**Results:**

A total of 15,233 patients underwent TAVR. The mean age was 81.5 ± 6.8 years, 56% were women, and Society of Thoracic Surgeons Predicted Risk of Mortality was 4.8% (interquartile range [IQR] 3.0-8.0%). Of these, 2731 (17.9%) patients had low-gradient AS with preserved or impaired LVEF, 138 (0.9%) discordant high-gradient AS, and 12,364 (81.2%) concordant high-gradient AS. There was no difference in 1-year mortality between discordant AS and concordant high-gradient AS (13.1 vs. 11.9%, adjusted hazard ratio 1.19, *p* = 0.08). One-year mortality rates was higher in low-gradient AS with impaired LVEF compared to concordant high-gradient AS (15.9 vs. 11.9%, adjusted hazard ratio 1.43, *p* = 0.01). Patients with concordant high-gradient AS had higher major bleeding rates (6.7%) compared to both low-gradient AS with impaired LVEF (4.0%) and preserved LVEF (5.4%) (*p* < 0.001 and *p* = 0.04).

**Conclusions:**

Nearly 20% of patients undergoing TAVR had discordant AS. One-year mortality was higher in low-gradient AS with impaired LVEF, whereas outcomes were similar among low-gradient with preserved LVEF, discordant high-gradient, and concordant high-gradient AS.

## Highlights

• The global Cerebrovascular Events in Patients Undergoing Transcatheter Aortic Valve Implantation 2 study reveals that nearly 20% of patients undergoing transcatheter aortic valve replacement do not exhibit the classical concordant high-gradient aortic stenosis (AS) profile.
• Patients with low-gradient AS with impaired left ventricular ejection fraction experienced the highest 1-year mortality rate.
• Low-gradient AS with preserved left ventricular ejection fraction, discordant high-gradient AS, and concordant high-gradient AS had similar 1-year survival.
• Bleeding after transcatheter aortic valve replacement was most frequent in patients with concordant high-gradient AS.

## Graphical abstract

**Figure:**
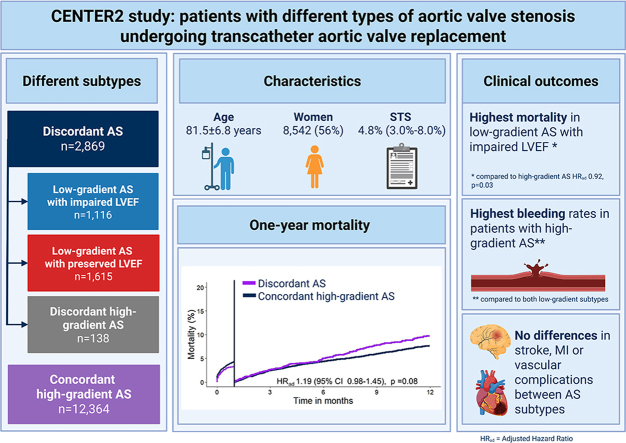

## Introduction

Transcatheter aortic valve replacement (TAVR) has revolutionized the treatment of patients with severe aortic stenosis (AS) across a broad range of indications.^1^ The high potential of TAVR was established in patients with “classical” concordant high-gradient AS (mean gradient ≥40 mmHg and aortic valve area [AVA] ≤ 1.0 cm^2^). However, in clinical practice, a growing number of patients referred for TAVR does not present with concordant echocardiographic characteristics of AS. Up to 50% of patients with severe AS have low gradients.^2^, ^3^, ^4^ Likewise, 3% to 10% of patients with high aortic gradients have an AVA greater than 1.0 cm^2^.^5^^,^^6^ Patients with those different types of AS do not meet the classical concordant criteria for intervention and were either excluded or represented only a small subset in the pivotal trials.^7^^,^^8^ Observational studies present conflicting results in patients with discordant AS, but data from a large worldwide cohort are missing.^6^^,^^9^, ^10^, ^11^, ^12^, ^13^ Therefore, these patients are challenging for multidisciplinary heart teams, as the optimal treatment of these patients is still to be elucidated. Considering the procedural risk of TAVR and the expected expansion of indications for TAVR, management of these challenging patients with discorant AS should be guided by epidemiological data on incidence and clinical outcomes. Our study aims to investigate the incidence and clinical outcomes in a real-world population of patients with different types of AS undergoing TAVR.

## Methods

The data that support the findings of this study are available from the corresponding author on reasonable request. The Cerebrovascular Events in Patients Undergoing Transcatheter Aortic Valve Implantation (CENTER) study is a worldwide, multicenter collaboration comprising 10 clinical studies including four single-center prospective registries, three national registries, two multicenter prospective registries, and one randomized clinical trial. The initial CENTER-study included 12,381 patients, and its design has been previously described in detail.^14^ Recently, collaborators were asked to add more contemporary patients; this led to the addition of 13,390 patients to the current CENTER2-database.^15^ The updated CENTER2 consists of 25,771 patients undergoing TAVR between 2007 and 2022. The database includes patient characteristics, echocardiographic data, procedural information, and long-term follow-up. Indication for TAVR and selection of transcatheter heart valve were made by the multidisciplinary heart team of each participating center.

### Patient Population

**Figure 1:**
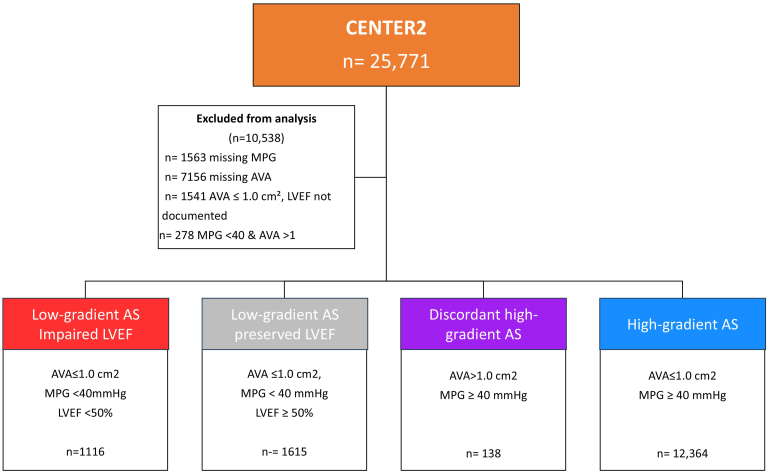
Patient population undergoing transcatheter aortic valve replacement divided into 4 subtypes. Abbreviations: AS, aortic stenosis; AVA, aortic valve area; CENTER, Cerebrovascular Events in Patients Undergoing Transcatheter Aortic Valve Implantation; LVEF, left ventricular ejection fraction; MPG, mean aortic pressure gradient.

Patients were included in the current study if they could be categorized in one of the four AS subtypes based on the baseline AVA, mean pressure gradient (MPG), and left ventricular (LV) ejection fraction (LVEF). Patients were categorized according to echocardiographic parameters into concordant or discordant criteria. For descriptive purposes, discordant AS comprised three subgroups, reflecting the absence of concordance between AVA and MPG, whereas the remaining group was classified as concordant high-gradient AS. All subgroups were described and analyzed separately. Concordant AS was defined as concordant high-gradient AS (AVA ≤1.0 cm^2^ and MPG ≥40 mmHg). As shown in Figure 1, the three discordant AS subgroups were divided as follows: 1) low-gradient AS with impaired LVEF (AVA ≤1.0 cm^2^, MPG <40 mmHg, and LVEF <50%), 2) low-gradient AS with preserved LVEF (AVA ≤1.0 cm^2^, MPG <40 mmHg, and LVEF ≥50%), and 3) and discordant high-gradient AS (AVA>1.0 cm^2^ and MPG ≥40 mmHg). Of the 25,771 patients enrolled in the CENTER2 registry, 10,538 were excluded due to missing key echocardiographic parameters (n = 1563 missing MPG; n = 7156 missing AVA; n = 1,541 missing LVEF) or discordant criteria (n = 278 with MPG <40 mmHg and AVA >1.0 cm^2^); this resulted in a final study population of 15,233 patients (Figure 1). Among these, 11.5% had missing survival status or incomplete follow-up and were therefore excluded from survival analyses. Stroke volume index, computerized tomography calcium scans, and stress echocardiography were not used for classification of the subgroups.

### Study Outcomes and Definitions

The primary outcomes were the incidence of discordant AS and all-cause mortality of patients undergoing after TAVR at 1-year follow-up. Other clinical outcomes included myocardial infarction, stroke, bleeding, and vascular complications. All events were defined according to the criteria of the second Valve Academic Research Consortium (VARC-2).^16^ The endpoints were defined according to VARC-2 criteria, as the study was initiated during the transition period to VARC-3, and the majority of participant enrollment occurred when VARC-2 was the prevailing standard. Although VARC-3 introduces updates and refinements to event definitions, most primary endpoints such as mortality, stroke, major vascular complications, and permanent pacemaker implantation remain largely consistent between the two versions. Secondly, the endpoint assessment was based on site-reported outcomes without centralized adjudication. Implanted valves were considered as newer generation if they became commercially available after 2013.

### Statistical Analysis

Continuous data are presented as mean with standard deviation (SD) or median with interquartile range (IQR), depending on distribution normality. Categorical data are presented as frequencies and percentage. Pearson chi-square test was performed to assess differences in clinical outcomes between the patient groups. As appropriate, continuous outcomes were analyzed using unpaired t-test or Mann–Whitney U test. The continuous data among the four groups were analyzed with a one-way analysis of variance. For data that was not normally distributed, the Kruskal–Wallis test was used. All baseline characteristics were explored as predictors for 1-year mortality using univariate Cox regression analysis. If *p* < 0.10 in the univariate model, the variable was incorporated into a multivariable Cox regression analysis. The multivariable Cox regression was performed after adjusting for age, gender, history of myocardial infarction, history peripheral vascular disease, baseline atrial fibrillation, estimated glomerular filtration rate lower than 30 mL/min/1.73 m^2^, and early-generation valves. The reference category for the adjusted hazard ratio (HR_ad_) and odds ratio was patients with concordant high-gradient AS. For analyses of the proportion of patients treated within each AS subgroup, data from the Spanish TAVI Registry were used. Temporal trends were assessed using logistic regression. Sensitivity analyses were conducted where patients with discordant high-gradient AS were excluded from the discordant AS group in the multivariable Cox regression analysis to explore potential heterogeneity.

We only included patients with new-generation valves. All *p* values were two-sided, and values of <0.05 were considered statistically significant. Calculations were performed using IBM SPSS Statistics (version 28.0 for Windows; SPSS, Inc., Chicago, Illinois).

## Results

**Supplemental Figure S1:**
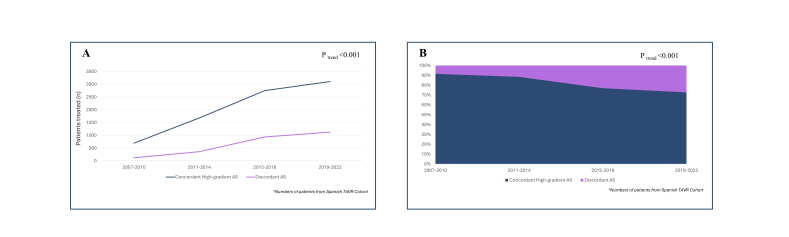
Proportion and trend of patients with different AS subgroups treated with TAVR (A) The number of patients undergoing TAVR in each AS subgroup over time, showing the trends in classical concordant high-gradient AS (blue) and discordant AS (purple) from 2007 to 2022 in the Spanish TAVI cohort. (B) The proportion of patients undergoing TAVR in the Spanish TAVI cohort, displaying the shift in the distribution of AS subgroups over the years Abbreviations: AS, aortic stenosis; TAVR, transcatheter aortic valve replacement.

A total of 15,233 patients were included in the current study. The mean age was 81.5 ± 6.8 years, 56% of patients were woman, and Society of Thoracic Surgeons Predicted Risk of Mortality score was 4.8% (IQR 3.0-8.0%). Of 2859 patients (18.8%) with discordant AS, 1116 patients had low-gradient AS with impaired LVEF (7.3%), 1,615 patients had low-gradient AS with preserved LVEF (10.6%), and 138 patients had discordant high-gradient AS (0.9%). A total of 12,364 patients were included with concordant high-gradient AS (81.2%). Over the study period, the proportion of patients with discordant AS increased from 15% in 2007–2011 to 27% in 2018–2022 (p_trend_<0.001, Supplemental Figure S1). Patients with discordant AS were younger (80.6 vs. 81.6 years, *p* < 0.001), were more often men (55.2 vs. 41.3%, *p* < 0.001), and had lower Society of Thoracic Surgeons Predicted Risk of Mortality scores (4.5 vs. 4.9%, *p* < 0.001) compared to concordant high-gradient AS. The median follow-up was 365 (IQR 46-685) days. Baseline characteristics of discordant AS and concordant high-gradient AS are shown in Table 1. Patients with concordant high-gradient AS had the lowest use of baseline antithrombotic therapy, with lower rates of dual antiplatelet therapy (28.4 vs. 50.5%, *p* < 0.001) and lower rates of oral anticoagulation (2.3 vs. 4.2%, *p* < 0.001) compared with discordant AS.

**Table 1:** Baseline and procedural characteristics in patients undergoing transfemoral transcatheter aortic valve replacement

	Discordant AS n = 2869	Concordant high-gradient AS n = 12,364	*p* value	
Demographics				
Age, y	80.6 ± 6.7	81.6 ± 6.5	<0.001	
Sex, female	1284 (44.8)	7258 (58.7)	<0.001	
BMI, kg/m^2^	27.0 ± 4.8	27.4 ± 4.9	<0.001	
Medical History				
Myocardial infarction	508 (27.0)	1371 (11.4)	<0.001	
Previous PCI	735 (23.2)	2437 (20.2)	<0.001	
Previous CABG	312 (11.5)	847 (7.9)	<0.001	
Hypertension	2403 (84.1)	9977 (80.9)	<0.001	
Peripheral vascular disease	483 (17.3)	2407 (13.6)	<0.001	
Diabetes mellitus	1172 (41.2)	3999 (32.5)	<0.001	
Dyslipidemia	1813 (63.8)	10,474 (58.7)	<0.001	
Previous cerebrovascular event	341 (12.0)	1304 (10.6)	0.10	
Permanent pacemaker	318 (11.7)	533 (7.0)	<0.001	
Atrial fibrillation	939 (33.6)	3068 (25.1)	<0.001	
Renal failure	299 (12.7)	1358 (12.3)	0.68	
NYHA functional class ≥3	1459 (53.8)	5078 (53.6)	0.82	
Risk scores				
Logistic EuroSCORE, %	14.8 (9.0-24.0)	12.8 (8.4-20.3)	<0.001	
EuroSCORE II, %	4.2 (2.6-7.5)	3.5 (2.2-5.6)	<0.001	
STS-PROM, %	4.5 (2.9-7.2)	4.9 (3.2-8.5)	<0.001	
Echocardiographic parameters				
Aortic valve area. cm^2^	0.74 ± 0.19	0.64 ± 0.17	<0.001	
Max gradient, mmHg	55.8 ± 13.2	86.7 ± 19.3	<0.001	
Mean gradient, mmHg	32.3 ± 7.5	54.2 ± 12.8	<0.001	
LVEF, %	51.3 ± 15.7	59.4 ± 11.4	<0.001	
Procedural parameters				
Transfemoral access	1009 (90.4)	11,804 (95.5)	<0.001	
Self-expandable valve, design:	1627 (56.7)	7128 (57.7)	0.37	
Supra-annular	1429 (87.9)	6605 (92.7)		
Intra-annular	197 (12.1)	522 (7.3)		
Balloon-expandable valve	1240 (43.3)	5234 (42.3)	0.37	
New generation valve	2177 (76.0)	6613 (53.6)	<0.001	
Valve-in-valve	54 (2.2)	146 (1.3)	<0.001	
Valve size, mm	26.7 ± 2.9	26.4 ± 2.7	<0.001	
Predilatation	1068 (42.5)	5356 (59.2)	<0.001	
Postdilatation	458 (16.9)	2460 (24.4)	<0.001	

Values are mean ± SD, n (%) or median (Q1-Q3). *p* value represents significance of the differences observed among the four types of aortic stenosis.

Abbreviations: AS, aortic stenosis; BMI, body mass index; CABG, coronary artery bypass graft; EuroSCORE, European System for Cardiac Operative Risk Evaluation; LVEF, left ventricle ejection fraction; NYHA, New York Heart Association; PCI, percutaneous coronary intervention; STS-PROM, Society of Thoracic Surgeons Predicted Risk of Mortality.

Patients with low-gradient AS and impaired LVEF had a higher prevalence of prior myocardial infarction (26.1 vs. 11.4%, *p* < 0.001), prior percutaneous coronary intervention (31.1 vs. 20.2%, *p* < 0.001), dyslipidemia (64.5 vs. 58.7%, *p* < 0.001), and New York Heart Association class III/IV (59.1 vs. 53.6%, *p* < 0.001) compared to concordant high-gradient AS. Baseline characteristics according to AS subtype are shown in Supplemental Table S1.

### Mortality

**Figure 2:**
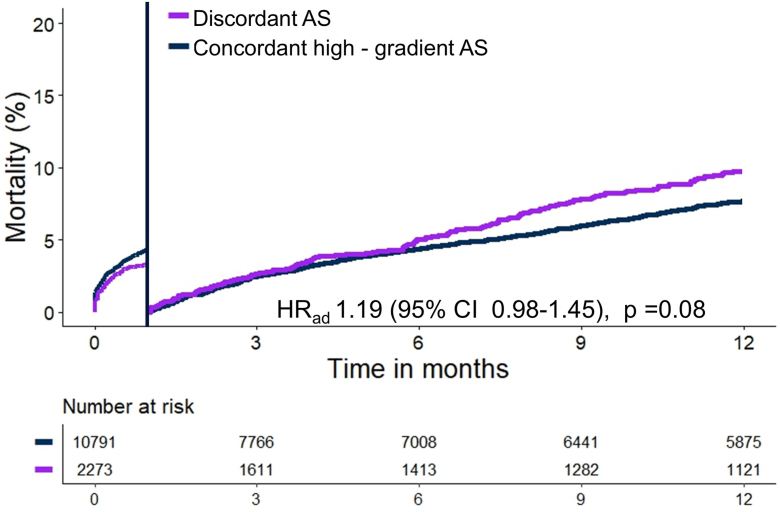
**Time-to-mortality curve for patients undergoing TAVR with low-gradient or discordant AS and classical high-gradient AS.** Time-to-mortality curve with landmark at 30 days for patients undergoing TAVR with discordant AS and concordant high-gradient AS. The adjusted hazard ratio is derived from multivariable Cox regression and represents the risk of mortality from 30 days to 1 year. One-year follow-up was complete in 57.4% of patients. Abbreviations: AS, aortic stenosis; HR_ad_, adjusted hazard ratio; TAVR, transcatheter aortic valve replacement.

Mortality did not differ between patients with discordant AS and concordant high-gradient at 30 days (3.5 vs. 4.3%, HR_ad_ 0.96, 95% CI 0.75-1.24, *p* = 0.78) or between 30 days to 1 year (13.1 vs. 11.9%, HR_ad_ 1.19, 95% CI 0.98-1.45, *p* = 0.08), Table 2 and Figure 2.

**Table 2:** Primary and secondary outcomes of the 4 subtypes of AS undergoing TAVR

	Group		HR_ad__j_ (95% CI)
	Discordant AS	Concordant high-gradient AS	Discordant AS vs. concordant high-gradient AS
	n = 2,869	n = 12,364	
Mortality			
30-d	96 (3.5 [2.8-4.2])	527 (4.3 [4.0-4.7])	0.96 (0.75-1.24)
1-year	253 (13.1 [11.5-14.6])	1145 (11.9 [11.2-12.6])	1.19 (0.98-1.45)
Clinical outcomes			OR (95% CI)
In-hospital permanent pacemaker implantation	378 (15.8)	1168 (16.5)	0.91 (0.81-1.02)
30-d myocardial infarction	22 (0.9)	126 (1.2)	0.76 (0.48-1.20)
30-d stroke	60 (2.1)	290 (2.3)	0.90 (0.67-1.18)
30-d major bleeding	144 (5.0)	805 (6.7)	0.73 (0.61-0.88)
30-d major vascular complication	16 (0.6)	77 (1.0)	0.57 (0.33-0.98)

Numbers are n (cumulative incidence [95% CI]) or n (%). The adjusted hazard ratio represents the risk of mortality from baseline to 30 and 30 d to 1 year. The reference group is concordant high-gradient AS. Thirty-day follow-up was complete in 85.7% of patients. One-year follow-up was complete in 57.4% of patients. Two-year follow-up was complete in 32.6%.

Abbreviations: AS, aortic stenosis; HR_ad__j_, adjusted hazard ratio; OR, odds ratio; TAVR, transcatheter aortic valve replacement.

**Figure 3:**
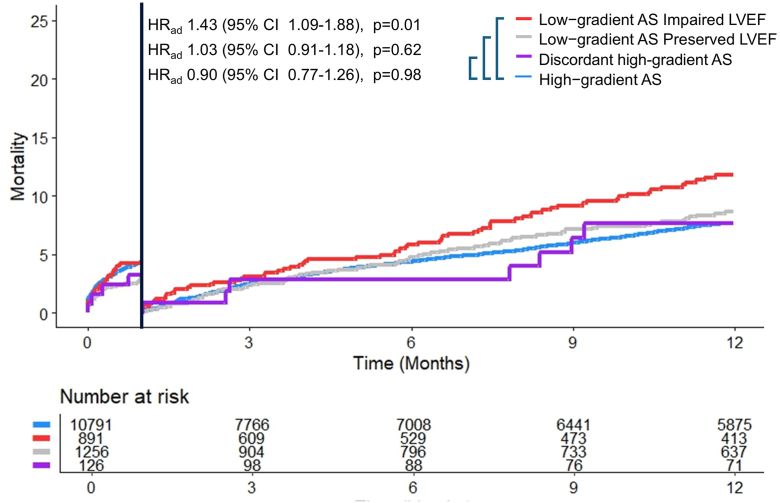
**Time-to-mortality curve for patients undergoing TAVR across thefoursubgroups.** Time-to-mortality curve with landmark at 30 days for patients undergoing TAVR in four subgroups. The adjusted hazard ratio is derived from multivariable Cox regression and represents the risk of mortality from 30 days to 1 year. One-year follow-up was complete in 57.4% of patients. Abbreviations: AS, aortic stenosis; HR_ad_, adjusted hazard ratio; LVEF, left ventricle ejection fraction; TAVR, transcatheter aortic valve replacement.

**Supplemental Figure S2:**
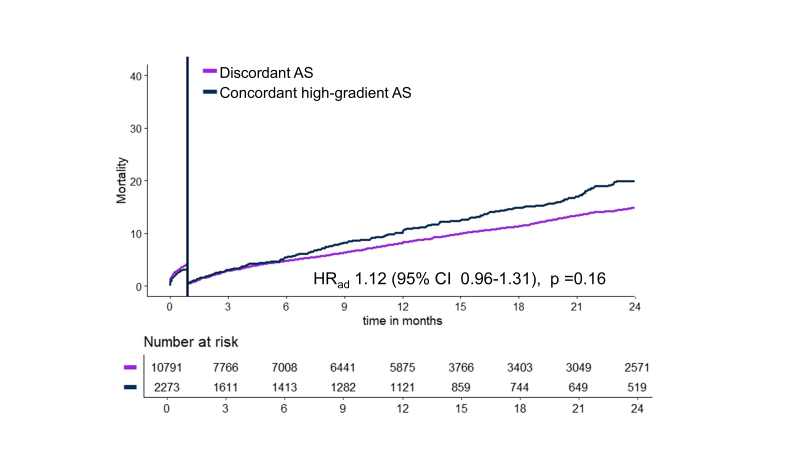
Two-year time to mortality curve for patients undergoing TAVR. Time to mortality curve with landmark at 30 days for patients undergoing TAVR between patients with discordant AS and classical concordant high-gradient AS. Adjusted hazard ratio represents patients undergoing TAVR with follow-up time over 30 days corrected for age, gender, baseline atrial fibrillation, history of myocardial infarction, peripheral vascular disease, chronic kidney dysfunction, and use of early generation valves Abbreviations: AS, aortic stenosis; HR_ad_, adjusted hazard ratio; TAVR, transcatheter aortic valve replacement.

**Supplemental Figure S3:**
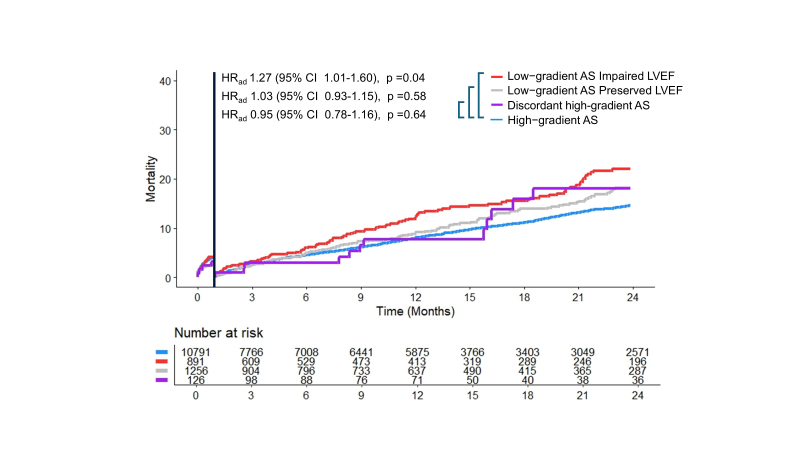
Two-year time to mortality curve for patients undergoing TAVR across the 4 subgroups. Time to mortality curve with landmark at 30 days for patients undergoing TAVR in 4 subgroups. Adjusted hazard ratio represents patients undergoing TAVR with follow-up time over 30 days corrected for age, gender, baseline atrial fibrillation, history of myocardial infarction, peripheral vascular disease, chronic kidney dysfunction, and use of early generation valves Abbreviations: AS, aortic stenosis; LVEF, left ventricle ejection fraction; TAVR, transcatheter aortic valve replacement; HR_ad_, adjusted hazard ratio.

Periprocedural and 30-day mortality were similar across the 4 types of AS. At 1 year, mortality rates were higher in patients with low-gradient AS with impaired LVEF compared to concordant high-gradient AS (15.9 vs. 11.9%, HR_ad_ 1.43, 95% CI 1.09-1.88, *p* = 0.01). There was no difference in 30-day to 1-year mortality rate between patients with low-gradient AS with preserved LVEF compared to concordant high-gradient AS (11.4 vs. 11.9%, HR_ad_ 1.03, 95% CI 0.91-1.18, *p* = 0.62). There was no difference in 30-day to 1-year mortality rate between patients with discordant high-gradient compared to concordant high-gradient AS (10.1 vs. 11.9%, HR_ad_ 0.98, 95% CI 0.77-1.26, *p* = 0.90). Figure 3 displays the 1-year mortality between four types of AS. The 2-year mortality is presented in Supplemental Figure S2 and S3.

### Clinical Outcomes

Major bleeding occurred in 949 (6.4%) patients. There was a higher incidence of major bleeding in patients with high-gradient AS (6.7%) compared to low-gradient AS with impaired LVEF (4.0%) and low-gradient AS with preserved LVEF (5.4%) (*p* < 0.001 and *p* = 0.04, respectively). Per 10 mmHg increase in the mean gradient, the risk of major bleeding increased by 1.08 (95% CI 1.03-1.13, *p* < 0.001). In-hospital permanent pacemaker implantation was not different between patients with low-gradient AS with impaired LVEF (14.9%), low-gradient AS with preserved LVEF (16.4%), discordant high-gradient AS (15.3%), or concordant high-gradient AS (16.5%) (*p* = 0.18). There was no difference in rates of myocardial infarction between patients with low-gradient AS with impaired LVEF (0.9%), low-gradient AS with preserved LVEF (1.0%), discordant high-gradient AS (0.9%), and concordant high-gradient AS (1.2%) (*p* = 0.88). There was no difference in stroke rates between patients with low-gradient AS with impaired LVEF (1.7%), patients with low-gradient AS with preserved LVEF (2.4%), and patients with concordant high-gradient AS (2.3%) (*p* = 0.43). There was no difference in vascular complications between patients with low-gradient AS with impaired LVEF (0.6%), low-gradient AS with preserved LVEF (0.5%), discordant high-gradient AS (1.6%), and patients with concordant high-gradient AS (1.0%) (*p* = 0.17). Table 2 and Supplemental Table S2 present clinical outcomes according to the VARC-2 across the subtypes.

### Sensitivity Analyses

All-cause mortality was similar in the sensitivity analysis that compared both subgroups of low-gradient AS with high-gradient AS. In an additional sensitivity analysis restricted to patients treated with new generation valves between 2018 and 2022, a total of 4,422 patients underwent TAVR. One-year mortality was higher in patients with low-gradient AS and impaired LVEF (17.4%) compared with concordant high-gradient AS (12.7%) (HR_ad__j_ 1.43, 95% CI 1.09-1.87, *p* = 0.010). One-year mortality did not differ between patients with low-gradient AS and preserved LVEF (10.6%) and concordant high-gradient AS (12.7%) (HR 1.03, 95% CI 0.91-1.18, *p* = 0.62). No difference in 1-year mortality was observed between patients with discordant high-gradient AS (26.7%) and concordant high-gradient AS (12.7%) (HR 0.98, 95% CI 0.77-1.26, *p* = 0.90).

## Discussion

This large, real-world study of patients undergoing TAVR showed that 1) almost 20% of patients undergoing TAVR had discordant AS; 2) patients presenting with low-gradient AS with impaired LVEF had higher 1-year mortality rates, while similar mortality rates were observed across other AS subtypes; and 3) patients with concordant high-gradient AS experienced the highest 30-day major bleeding.

### Incidence of Discordant AS

Our study showed that Heart Teams are increasingly confronted with patients with AS who do not meet the classical echocardiographic criteria for severe disease. Our findings demonstrate that discordant AS occurs in approximately one in five patients undergoing TAVR. This is consistent with current literature, which reports discordant high-gradient AS in around 6% to 10%, whereas low-gradient AS has been reported in up to one-third of patients undergoing TAVR.^6^^,^^9^^,^^16^^,^^17^ Determining the indication for valve replacement remains challenging in patients with discordant AS, as standard echocardiographic markers provide conflicting information regarding disease severity. In this setting, the inconsistency between the aortic valve gradient and AVA limits diagnostic certainty and complicates risk stratification.

### Low-Gradient AS

Patients with low-gradient AS encompass the largest proportion of patients with discordant AS, and management in this group remains challenging for Heart Teams. Patients with low-gradient AS with reduced LVEF are a distinct subgroup with typically a high-risk profile as our study showed that patients with low-gradient AS are characterized by higher rates of ischemic heart disease, peripheral vascular disease, and presentation with more advanced symptoms as indicated by higher New York Heart Association class. Consistent with prior reports, in our study, all-cause mortality was higher in patients with low-gradient AS with impaired LVEF.^9^^,^^17^

LV dysfunction in these patients reflects the combined effects of ischemic myocardial injury and chronic pressure overload imposed by the stenotic aortic valve. As a result, low-gradient AS with impaired LVEF is particularly challenging for multidisciplinary Heart Teams; LV function may improve after valve replacement when systolic dysfunction is predominantly driven by excessive afterload, but recovery is less likely when the myocardial fibrosis is related to prior myocardial infarction.^1^ The extent of cardiac damage has been shown to be independently associated with increased 2-year mortality after TAVR.^18^

Importantly, in a study by Genereux et al.^19^, 85% of patients undergoing TAVR had either no change or deterioration in cardiac damage at 1 year after the procedure. The lack of improvement in cardiac function postaortic valve replacement suggests patients may be treated too late. Treating patients earlier before the cardiac damage accumulates has the potential to minimize cardiac remodeling. This concept is supported by our findings, which show that patients with low-gradient AS with preserved LVEF have outcomes comparable to those with classical concordant high-gradient AS.

The TAVR Unload evaluated whether TAVR provides clinical benefit in patients with moderate AS and reduced LVEF. However, it failed to demonstrate an improvement in the primary hierarchical composite endpoint. The study enrolled only 178 of the planned 600 patient patients and required multiple protocol modifications, which necessitate cautious interpretation of the overall findings.^20^ The PROGRESS [NCT04889872] and EXPAND TAVR II [NCT05149755] will provide further guidance in determining the optimal timing of TAVR in patients with moderate AS.

### Discordant High-Gradient

Discordant high-gradient AS represents a complex subgroup, sharing several of the challenges observed in low-gradient AS. Recognition of these discordant AS subgroups is important for Heart Teams when echocardiographic parameters fall within the range of moderate AS. In our study, patients with discordant high-gradient AS were younger, had fewer comorbidities, and had lower surgical risk scores than other subgroups. Despite these apparently favorable baseline characteristics, mortality rates were comparable to those observed in patients with concordant high-gradient AS. Prior studies have suggested that the combination of elevated gradients and an AVA greater than 1.0 cm^2^ is often related to high flow states, larger body size, and larger LV outflow tract dimensions rather than less severe valvular obstruction.^6^^,^^16^ Although flow and LV outflow tract measurements were not available in the present study, the comparable mortality observed across high-gradient phenotypes suggests that elevated transvalvular gradients may reflect disease severity and clinical risk beyond valve area alone; this underscores the need for careful interpretation of discordant echocardiographic findings.

### Major Bleeding

Major bleeding was more frequent among patients with concordant high-gradient AS. This is of particular importance because bleeding remains a clinically relevant complication with increased mortality rates and should be anticipated in patients with high gradients undergoing TAVR.^21^ Interestingly, we found that major bleeding risk increased incrementally with higher mean transvalvular gradients. This may be due to the correlation between the pressure valve gradient and the severity of von Willebrand factor (vWF) abnormalities. High shear stress around the stenotic aortic valve in patients with high-gradient AS unfolds the vWF protein, which enables ADAMTS13 to cleave the protein into various sizes.^22^^,^^23^ Although vWF levels increase within minutes after aortic valve replacement, with full recovery in 86% of patients within the first day, the high rate of proteolysis before intervention may explain the increased bleeding risk observed in patients with high-gradient AS, as only the larger multimers of vWF are hemostatically active.

### Future Perspectives

The findings of this real-world study show that a substantial proportion of patients undergoing TAVR do not comply with the standard echocardiographic characteristics of classical concordant high-gradient AS. Improved recognition and characterization of discordant AS has the potential to directly improve patient care by supporting more accurate risk stratification, timelier referral, and individualized treatment decisions and thereby reduce delays in intervention and preventing progression to irreversible cardiac damage. Although mortality remains an important and informative endpoint, it does not fully capture the broader clinical impact of the disease, such as patient-centered aspects of symptom burden and quality of life. Unfortunately, our current data set's limited ability to include these broader measures was a significant limitation. Future studies should aim to incorporate standardized,patient-reported outcome measures and functional assessments to better inform clinical decision-making. Nevertheless, our findings underscore the need for careful interpretation of discordant echocardiographic findings to avoid underestimation of disease severity and prognosis, which may contribute to delayed referral for valve replacement. Despite the increased use of TAVR, more than 40% of patients with severe AS remain untreated despite guideline-based indications.^24^^,^^25^ Reliance on a pressure gradient below 40 mmHg or an AVA > 1.0 cm^2^ may provide false reassurance, and earlier recognition may be required to prevent progression of cardiac damage.

## Study Limitations

The CENTER2 is an observational real-world study that comes with known limitations. With the inclusion of 10 centers, there is a potential for underreporting of clinical outcomes due to on-site reporting and absence of a central event adjudication process. Because the CENTER2 study did not routinely collect complete echocardiographic data of mean gradient, LV function, and AVA, not all patients from the overall study were eligible for inclusion in this current analysis; this may have impacted the presented results. In addition, more advanced diagnostic parameters, including stroke volume index, computerized tomography calcium scans, and stress echocardiography, were not routinely collected as recommended for diagnosing severe aortic valve stenosis by current guidelines. This omission may have resulted in the inclusion of patients with normal flow and low-gradient AS for whom guidelines suggest conservative management and the potential inclusion of patients with nonsevere AS within the low-gradient group. Therefore, our study reflects routine clinical practice rather than outcomes stratified by guideline-defined hemodynamic subtypes. The incomplete 1-year follow-up may introduce bias, although sensitivity analysis found no significant differences between patients with and without 1-year follow-up. Future studies should aim to incorporate more comprehensive data collection protocols to minimize such exclusions and enhance the robustness of subgroup analyses.

## Conclusion

In this large contemporary real-world study, almost 20% of patients undergoing TAVR did not fit the standard characteristics of concordant high-gradient AS. Patients with low-gradient AS with impaired LVEF had the highest 1-year mortality, whereas mortality rates were similar between low-gradient AS with preserved LVEF, discordant high-gradient AS, and concordant high-gradient AS. Moreover, high-gradient AS was associated with higher rates of major bleeding.

## Ethics Statement

All individual studies of the CENTER2 study were conducted according to the Declaration of Helsinki. All patients provided written informed consent at their local hospital. Institutional review board approval was obtained at each center. The study is registered at ClinicalTrials.gov (NCT03588247).

## Funding

Funding for this paper was provided by the Netherlands CardioVascular Research Initiative: the Dutch Heart Foundation (CVON 2018-28 & 2012-06 Heart Brain Connection), Dutch Federation of University Medical Centres, the Netherlands Organisation for Health Research and Development, and the Royal Netherlands Academy of Sciences. The study also received support from the Netherlands Organisation for Health Research and Development.

## Disclosure Statement

Fabio S. de Brito Jr is a proctor for Edwards Lifesciences and Medtronic. Marco Barbanti is consultant for Edwards Lifesciences and received speaker honoraria from Medtronic and Biotronik. Azeem Latib is a consultant for Medtronic and received honoraria from Abbott Vascular. Matteo Pagnesi has received personal fees from Abbott Vascular. Ronak Delewi received educational grants from Boston Scientific, Abiomed, Edwards Lifesciences, Sanofi, Meril life Science, Novartis, and Amgen.

The other authors had no conflicts to declare.
