# Supplementary material for: Different Types of Aortic Valve Stenosis in Patients Undergoing Transcatheter Aortic Valve Replacement
Source: Struct Heart. 2026 Feb 10;10(5):100817. doi: 10.1016/j.shj.2026.100817 (PMC12996641; doi:10.1016/j.shj.2026.100817)
Supplement: Supplemental Figures and Tables [file mmc1.docx]

**Supplemental Figure S1: Proportion and Trend of patients with different AS subgroups treated with TAVR**

*Legend:* ***A)*** *The number of patients undergoing TAVR in each AS subgroup over time, showing the trends in classical concordant high-gradient AS (blue) and discordant AS (purple) from 2007 to 2022 in the Spanish TAVI cohort.* ***B)*** *The proportion of patients undergoing TAVR in the Spanish TAVI cohort, displaying the shift in the distribution of AS subgroups over the years.*

**Supplemental Figure S2: Two-year Time to mortality curve for patients undergoing TAVR.**

*Legend: Time to mortality curve with landmark at 30 days for patients undergoing TAVR between patients with discordant AS and classical concordant high-gradient AS. Adjusted hazard ratio represents patients undergoing TAVR with follow-up time over 30 days corrected for age, gender, baseline atrial fibrillation, history of myocardial infarction, peripheral vascular disease, chronic kidney dysfunction, and use of early generation valves. AS= aortic stenosis; TAVR = transcatheter aortic valve replacement..*

**Supplemental Figure S3: Two-year time to mortality curve for patients undergoing TAVR across the four subgroups.**

*Legend: Time to mortality curve with landmark at 30 days for patients undergoing TAVR in four subgroups. Adjusted hazard ratio represents patients undergoing TAVR with follow-up time over 30 days corrected for age, gender, baseline atrial fibrillation, history of myocardial infarction, peripheral vascular disease, chronic kidney dysfunction, and use of early generation valves. AS= aortic stenosis; LVEF = left ventricle ejection fraction; TAVR = transcatheter aortic valve replacement.*

**Supplemental Table S1: Baseline and Procedural Characteristics in Patients Undergoing TAVR.**

|  |  |  |  |  |  |  |  |  |  |  |  |  |
| --- | --- | --- | --- | --- | --- | --- | --- | --- | --- | --- | --- | --- |
|  | **Low-gradient AS Impaired LVEF**  n=1,116 | | | **Low-gradient AS Preserved LVEF**  n=1,615 | | **Discordant**  **High-gradient AS**  n=138 | | | | **Concordant High-gradient AS**  n=12,364 | | **p Value** |
| Demographics |  | | |  | | |  | | | |  |  |
| Age, y | 79.2±7.2 | | | 81.7±5.9 | | | 79.1±7.7 | | | | 81.6±6.5 | <0.001 |
| Woman | 355 (31.8) | | | 869 (53.8) | | | 60 (43.5) | | | | 7258 (58.7) | <0.001 |
| BMI, kg/m^2^ | 27.2±4.7 | | | 27.7±4.8 | | | 27.4±6.6 | | | | 27.4 ±4.9 | <0.001 |
| Medical History |  | | |  | | |  | | | |  |  |
| Myocardial infarction | 278 (26.1) | | | 220 (11.7) | | | 10 (7.3) | | | | 1371 (11.4) | <0.001 |
| Previous PCI | 334 (31.1) | | | 385 (25.1) | | | 16 (11.7) | | | | 2437 (20.2) | <0.001 |
| Previous CABG | 154 (14.5) | | | 145 (9.6) | | | 13 (9.5) | | | | 847 (7.9) | <0.001 |
| Hypertension | 928 (83.2) | | | 1364 (84.9) | | | 111 (81.0) | | | | 9977 (80.9) | <0.001 |
| Peripheral vascular disease | 225 (20.5) | | | 235 (15.1) | | | 23 (16.8) | | | | 2407 (13.6) | <0.001 |
| Diabetes mellitus | 509 (45.9) | | | 621 (38.9) | | | 42 (30.9) | | | | 3999 (32.5) | <0.001 |
| Dyslipidemia | 717 (64.5) | | | 8.3 (63.7) | | | 10474 (58.7) | | | | 10474 (58.7) | <0.001 |
| Previous cerebrovascular events | 135 (12.2) | | | 185 (11.6) | | | 21 (15.3) | | | | 1304 (10.6) | 0.10 |
| Permanent pacemaker | 170 (15.6) | | | 145 (9.3) | | | 3 (4.8) | | | | 533 (7.0) | <0.001 |
| Atrial fibrillation | 3068 (25.1) | | | 363 (33.1) | | | 541 (34.6) | | | | 35 (25.5) | <0.001 |
| Renal failure | 122 (13.2) | | | 165 (12.6) | | | 12 (9.7) | | | | 1358 (12.3) | 0.68 |
| NYHA functional class ≥3 | 632 (59.1) | | | 775 (49.8) | | | 6484 (50.7) | | | | 5078 (53.6) | <0.001 |
| Risk scores |  | | |  | | |  | | | |  |  |
| Logistic EuroSCORE, % | 19.8 (12.5-32.0) | | | 12.1 (8.0-19.5) | | | 10.7 (6.5-18.0) | | | | 12.8 (8.4-20.3) | <0.001 |
| EuroSCORE II, % | 6.0 (3.6-11.0) | | | 3.6 (2.2-5.5) | | | 3.1 (2.3-4.5) | | | | 3.5 (2.2-5.6) | <0.001 |
| STS-PROM, % | 5.0 (3.1-8.1) | | | 4.3 (2.8-6.6) | | | 3.7 (2.4-6.3) | | | | 4.9 (3.2-8.5) | <0.001 |
| Echocardiographic parameters | | |  | |  | | |  |  |  |  |  |
| Aortic valve area, cm^2^ | 0.70 ±0.17 | | | 0.74±0.17 | | | 1.2 ±0.18 | | | | 0.64±0.17 | <0.001 |
| Max gradient, mmHg | 49.9±12.2 | | | 58.2±11.1 | | | 77.9±14.3 | | | | 86.7±19.3 | <0.001 |
| Mean gradient, mmHg | 29.2±6.7 | | | 33.0±5.8 | | | 48.3±8.7 | | | | 54.2±12.8 | <0.001 |
| LVEF, % | 34.6±8.5 | | | 62.5± 7.2 | | | 59.4±9.8 | | | | 59.4±11.4 | <0.001 |
| Procedural Parameters |  | | |  | | |  | | | |  |  |
| Transfemoral access | 1009 (90.4) | | | 1501 (92.9) | | | 135 (97.8) | | | | 11,804 (95.5) | <0.001 |
| Self-expandable valve | 593 (53.2) | | | 966 (59.8) | | | 67 (48.6) | | | | 7,127 (57.7) | <0.001 |
| Newer-generation Valve | 823 (74.8) | | | 1,285 (79.7) | | | 60 (43.5) | | | | 6,613 (53.6) | <0.001 |
| Valve size, mm | 27.3±2.9 | | | 26.4±2.8 | | | 26.3±2.9 | | | | 26.4±2.7 | <0.001 |
| Predilation | 370 (38.1) | | | 642 (44.6) | | | 56 (56.6) | | | | 5,356 (59.2) | <0.001 |
| Postdilatation | 206 (19.1) | | | 233 (15.2) | | | 19 (17.8) | | | | 2,460 (24.4) | <0.001 |

*Legend: Values are mean ±SD, n (%), or median (Q1-Q3). P-value represents significance of the differences observed among the four types of aortic stenosis. BMI= body mass index; CABG= coronary artery bypass graft; eGFR= estimated glomerular filtration rate; EuroSCORE= European System for Cardiac Operative Risk Evaluation; LVEF= Left ventricle ejection fraction; NYHA= New York Heart Association; PCI= percutaneous coronary intervention; and STS-PROM: Society of Thoracic Surgeons Predicted Risk of Mortality.*

**Supplemental Table S2: Primary and Secondary Outcomes in the Different Subgroups of AS**

|  |  | Group | | | HR_ad_ and P value | | |
| --- | --- | --- | --- | --- | --- | --- | --- |
|  | Low-gradient AS impaired LVEF | Low-gradient AS preserved LVEF | Discordant high-gradient AS | High-gradient AS | Low-gradient AS impaired LVEF  vs  Concordant  High-gradient AS | Low-gradient AS preserved LVEF  vs  Concordant  High-gradient AS | Discordant  high-gradient AS  vs  Concordant  High-gradient AS |
|  | n= 1,116 | n= 1,615 | n= 138 | n= 12,364 |  |  |  |
| Mortality |  |  |  |  |  |  |  |
| Thirty-day | 47 (4.9) | 45 (3.4) | 4 (3.1) | 527 (4.8) | 1.26 (0.89-1.79) | 0.90 (0.75-1.08) | 0.90 (0.65-1.25) |
| One-year | 119 (19.5) | 122 (13.9) | 12 (13.5) | 1145 (15.2) | 1.43 (1.09-1.88) | 1.03 (0.91-1.18) | 0.90 (0.77-1.26) |
| Two-years | 154 (43.5) | 170 (36.0) | 18 (31.6) | 1456 (34.3) | 1.27 (1.01-1.60) | 1.03 (0.93-1.15) | 0.95 (0.78-1.16) |
| Thirty-day Outcomes |  |  |  |  | **OR and P value** | | |
| Myocardial infarction | 8 (0.9) | 13 (1.0) | 1 (0.9) | 126 (1.2) | 0.69 (0.34-1.42) | 0.81 (0.46-1.44) | 0.75 (0.10-5.40) |
| Stroke | 19 (1.7) | 38 (2.4) | 3 (2.2) | 290 (2.3) | 0.72 (0.45-1.15) | 1.00 (0.71-1.41) | 0.93 (0.29-2.92) |
| Major bleeding | 45 (4.0) | 87 (5.4) | 12 (8.9) | 805 (6.7) | 0.58 (0.43-0.79) | 0.79 (0.63-0.99) | 1.35 (0.74-2.45) |
| Major Vascular Complication | 7 (0.6) | 8 (0.5) | 1 (1.6) | 77 (1.0) | 0.63 (0.29-1.36) | 0.50 (0.24-1.03) | 1.57 (0.21-11.43) |

*Legend: Numbers are n (%). Adjusted hazard ratio [HR_ad_] corrected for age, gender, baseline atrial fibrillation, history of myocardial infarction, peripheral vascular disease, chronic kidney dysfunction, and use of early generation valves. The reference group is concordant high-gradient AS. Thirty-day follow-up was complete in 85.7% of patients. One-year follow-up was complete in 57.4% of patients and two-year follow-up was complete in 32.6%. AS= aortic stenosis; LVEF = left ventricle ejection fraction.*
